# Genetic polymorphisms, efficiency and toxicity of cancer chemotherapy

**DOI:** 10.1186/1897-4287-13-S1-A5

**Published:** 2015-09-09

**Authors:** Karolina Tecza, Jolanta Pamula-Pilat, Zofia Kolosza, Natalia Radlak, Ewa Grzybowska

**Affiliations:** 1 Center for Translational Research and Molecular Biology of Cancer, Maria Sklodowska-Curie Memorial Cancer Center and Institute of Oncology, Gliwice Branch, Gliwice, Poland; 2 Department of Epidemiology and Silesia Cancer Registry, Maria Sklodowska-Curie Memorial Cancer Center and Institute of Oncology, Gliwice Branch, Gliwice, Poland; 3 Institute of Automatic Control, Silesian University of Technology, Gliwice, Poland

## 

Human reaction to drugs, including chemotherapeutics, is an extremely complex process, with many enzymes of different metabolic and signal transduction pathways involved. Despite the knowledge already gathered, the exact mechanisms responsible for individual patients’ responses to chemotherapy drugs, including adverse reactions, are still not clear. Cellular transport and detoxification systems evolved as natural protection against xeniobiotics and they process substrates that belong to diverse chemical classes. There is evidence that polymorphic variants in genes encoding proteins involved in influx, efflux and two metabolism phases (i.e. ABC and SLC transporters, CYP and GST enzymes family) can both influence chemotherapeutic regimes' effectiveness and toxicity. Also, because the drug-induced damages to nucleic acid structure are responsible for activating cell death programme, it is assumed that, apart from transport and detoxification systems, resistance to these drugs can also be the result of effective DNA repair systems, defective checkpoint activation and flawed recognition of DNA adducts. It is plausible that functional polymorphisms in genes encoding key proteins of said mechanisms (i.e. ERCC and XRCC families, p53, ATM) will influence cells’ sensitivity to drugs.

Chemotherapeutic regimes are usually composed of non-crossing agents aimed at a variety of cellular targets and processes in order to overcome cells' resistance to single drugs, for example 5-fluorouracil, doxorubicin and cyclophosphamide are used as FAC regime in breast cancer, or carbo/cisplatin administered with paclitaxel in treatment of ovarian cancer. This approach substantially increases the chance of success of a treatment, but also increases the number of factors potentially responsible for treatment failure and/or severe adverse reactions. Consistently, insensitivity or oversensitivity to multidrug treatment has to be a result of global changes in activity of proteins involved in every, or some of the mechanisms responsible for used drugs transport, metabolism, and their specific impact on the cell. For FAC, it has been shown that pyrimidine analog 5-fluorouracil's effectiveness could be modulated in the presence of polymorphic variants in SLC22, CYP, and GST gene families. Similarly to 5-fluorouracil, polymorphisms in genes for CYPs could be responsible for different cells’ reaction to cyclophosphamide, and reduced drug detoxification rates could be connected to functional variants in GST isoform genes. For the ovarian cancer paclitaxel/cisplatin regime there is strong evidence that accumulation of several polymorphic variants is responsible for elevated risk of death and progression (*PGR, ATM* and *ABCB1* gene variants). Such accumulation is also correlated with many symptoms of chemotherapy systemic toxicities, such as haematological - including neutropenia, gastro-intestinal and hepatological toxicity (polymorphisms in *ATP7B* and *PGR* genes).

The search for genetic factors connected to the high risk of treatment failure and severe toxicity has become one of the foundations of personalised medicine. Simultaneous analysis of many genetic factors, which individually may have very subtle impact on the cellular processes and therefore may be omitted, allows the understanding of their cumulative impact on the chemotherapy response. Insight into the complex molecular mechanisms resulting in drug resistance or oversensitivity during cancer treatment, may be potentially relevant for the further research focused on developing drugs tailored to cancer patients’ genetic profile.

